# Modulation of Motor Cortical Inhibition and Facilitation by Touch Sensation from the Glabrous Skin of the Human Hand

**DOI:** 10.1523/ENEURO.0410-23.2024

**Published:** 2024-03-01

**Authors:** Shancheng Bao, Yiyu Wang, Yori R. Escalante, Yue Li, Yuming Lei

**Affiliations:** ^1^Program of Motor Neuroscience, Department of Kinesiology & Sport Management, Texas A&M University, College Station, Texas 77843; ^2^Department of Neuroscience & Experimental Therapeutics, Texas A&M University, College Station, Texas 77843

**Keywords:** brain stimulation, finger stimulation, primary motor cortex, primary somatosensory cortex, the glabrous skin

## Abstract

Touch sensation from the glabrous skin of the hand is essential for precisely controlling dexterous movements, yet the neural mechanisms by which tactile inputs influence motor circuits remain largely unexplored. By pairing air-puff tactile stimulation on the hand's glabrous skin with transcranial magnetic stimulation (TMS) over the primary motor cortex (M1), we examined the effects of tactile stimuli from single or multiple fingers on corticospinal excitability and M1's intracortical circuits. Our results showed that when we targeted the hand's first dorsal interosseous (FDI) muscle with TMS, homotopic (index finger) tactile stimulation, regardless of its point (fingertip or base), reduced corticospinal excitability. Conversely, heterotopic (ring finger) tactile stimulation had no such effect. Notably, stimulating all five fingers simultaneously led to a more pronounced decrease in corticospinal excitability than stimulating individual fingers. Furthermore, tactile stimulation significantly increased intracortical facilitation (ICF) and decreased long-interval intracortical inhibition (LICI) but did not affect short-interval intracortical inhibition (SICI). Considering the significant role of the primary somatosensory cortex (S1) in tactile processing, we also examined the effects of downregulating S1 excitability via continuous theta burst stimulation (cTBS) on tactile–motor interactions. Following cTBS, the inhibitory influence of tactile inputs on corticospinal excitability was diminished. Our findings highlight the spatial specificity of tactile inputs in influencing corticospinal excitability. Moreover, we suggest that tactile inputs distinctly modulate M1's excitatory and inhibitory pathways, with S1 being crucial in facilitating tactile–motor integration.

## Significance Statement

Tactile sensations from the glabrous skin of the hand are crucial for controlling dexterous hand movements. This research delves into how tactile inputs modulate corticospinal excitability and motor circuits. By pairing air-puff tactile stimulation on single and multiple fingers with transcranial magnetic stimulation targeting the motor cortex, our findings highlight the spatial specificity of tactile inputs in shaping corticospinal excitability. Our results further suggest that tactile stimuli can distinctly influence the motor cortex's excitatory and inhibitory mechanisms. Finally, we propose that the somatosensory cortex plays an important role in facilitating tactile–motor integration.

## Introduction

Tactile feedback from the glabrous skin of the hand is crucial for fine motor control, grip adjustments, and dexterous manipulations. The low-threshold mechanoreceptors (LTMRs) innervating the glabrous skin capture abundant information about the mechanical properties and interactions of the objects we interact with, including the presence of contact with objects, the pressure exerted on objects, object texture and shape, object deformation, and the slippage of an object against the skin (Johansson and Vallbo, 1983; Johansson and Westling, 1984; Johansson and Flanagan, 2009; Zimmerman et al., 2014; Zangrandi et al., 2021). Such tactile information is utilized to regulate motor commands, ensuring the precise and efficient execution of movements. Lesion evidence involving monkeys revealed that, following hand deafferentation, the monkeys were incapable of executing fine hand movements (Mott and Sherrington, 1895). Additionally, after the temporary inactivation of area 3b—an area associated with processing tactile inputs—there were notable impairments in the monkeys’ capability to control finger movements during precision grips (Hikosaka et al., 1985; Brochier et al., 1999). In agreement, individuals with somatosensory impairments showed deficits in timing and force adjustments during grasping movements (Blennerhassett et al., 2007). Moreover, a person with complete somatosensory deafferentation has difficulty performing fine motor activities such as grasping, writing with a pen, or fastening shirt buttons (Rothwell et al., 1982). While it is widely accepted that the successful execution of voluntary movements is supported by the interactions between the somatosensory and motor systems (Prochazka et al., 1997; Desmurget and Grafton, 2000; Schieber, 2001; Davis et al., 2022), the neural mechanisms by which tactile inputs influence motor circuits in humans remain largely unexplored.

The structural foundation for somatosensory–motor interactions is rooted in the dense anatomical connections between S1 and M1 (White and DeAmicis, 1977; Donoghue and Parham, 1983; Veinante and Deschênes, 2003; Mao et al., 2011), which enable somatosensory signals to effectively modulate M1 activity (Tokimura et al., 2000; Lei and Perez, 2017). Anatomical studies using injected tracers have identified direct neuron projections from S1 to M1 in rodents (Bedwell et al., 2014) and primates (Krubitzer and Kaas, 1990; Stepniewska et al., 1993). Electrophysiological research has indicated that direct stimulation of S1 elicits responses in M1 (Osborn et al., 2021) and movement-associated activities in S1 can be inferred from preceding M1 activities (Umeda et al., 2019). Imaging research has revealed U-shaped fibers linking the homuncular representations of S1 and M1 (Catani et al., 2012). Additionally, somatosensory stimulation triggers a BOLD response initially in the ventral posterolateral nucleus (VPL), then in S1, followed by M1 (Jung et al., 2021). Afferent inhibition serves as a prominent electrophysiological technique to delve into somatosensory–motor interactions (for a review, see Turco et al., 2018). This method is characterized by the suppression of corticospinal excitability triggered by somatosensory stimuli, achieved by coupling somatosensory stimulation with transcranial magnetic stimulation (TMS). The effects of tactile inputs on M1's intracortical excitatory and inhibitory circuitry can be assessed by applying a tactile stimulus followed by paired-pulse TMS over M1 (Sailer et al., 2002). Here, we integrated air-puff tactile stimulation combined with either single- or paired-pulse TMS to examine how tactile inputs from the glabrous skin of the hand influence corticospinal excitability and M1's intracortical circuits.

A critical question to address is: How does stimulation of homotopic versus heterotopic fingers, or simultaneous stimulation of multiple fingers, influence the modulation of corticospinal excitability of the hand muscles? Additionally, do tactile inputs from finger areas with a higher innervation density result in a more pronounced modulation of corticospinal excitability compared with areas with a lower innervation density? In Experiment 1, we applied TMS to the first dorsal interosseous (FDI) muscle of the hand while tactile stimuli were administered to either the homotopic (index) finger, the heterotopic (ring) finger, or all five fingers simultaneously. Evidence suggests that the fingertip possesses the highest innervation density compared with the finger's base (Corniani and Saal, 2020). We also explored the differential effects of stimulating these two regions of the index finger on corticospinal excitability of the FDI muscle. Prior research indicates that the neuronal pathways facilitating somatosensory–motor interactions may be mediated via intracortical inhibitory and facilitatory circuits in M1 (Sailer et al., 2002). In Experiments 2–4, we delved into how tactile inputs from the glabrous skin of the hand affect the three distinct intracortical mechanisms in M1: intracortical facilitation (ICF), short-interval intracortical inhibition (SICI), and long-interval intracortical inhibition (LICI). These mechanisms can be respectively interpreted to shed light on glutamate-driven excitatory functions (Ziemann et al., 1998; Schwenkreis et al., 1999), GABA_A_ receptor activities (Kujirai et al., 1993; Ilić et al., 2002), and GABA_B_ receptor activities in M1 (Valls-Solé et al., 1992; Wassermann et al., 1996). Considering the significant role of S1 in tactile processing (Romo et al., 2012) and the capability of continuous theta burst stimulation (cTBS) to produce a sustained suppression of S1 (Jacobs et al., 2014), we further probed the effect of S1 on tactile–motor interactions in Experiment 5.

## Materials and Methods

### Participants

The study included 31 right-handed, healthy participants, consisting of 19 females and 12 males. All participants were naive to the paradigm and the purpose of the study. All experimental protocols were approved by the Institutional Review Board of Texas A&M University. All participants gave written informed consent prior to participation, which was approved by the local ethics committee at Texas A&M University in accordance with the Declaration of Helsinki. Of the total participants, 20 were involved in the main five experiments, while an additional five were added to the control experiments due to some initial participants’ unavailability for subsequent testing.

### Electromyographic (EMG) recordings

Surface EMG was recorded from the FDI muscles through disposable Ag-AgCl surface electrodes with a diameter of 10 mm secured to the skin over the belly of the muscle. The acquired EMG signals were amplified and filtered (bandwidth, 30–2,000 Hz) with a bioamplifier (Neurolog System, Digitimer). These signals were then digitized at a sampling rate of 10 kHz via a CED Micro 1401 A/D converter (Cambridge Electronic Design) and stored on a computer for offline analysis.

### Tactile stimulation

**Figure 1.**
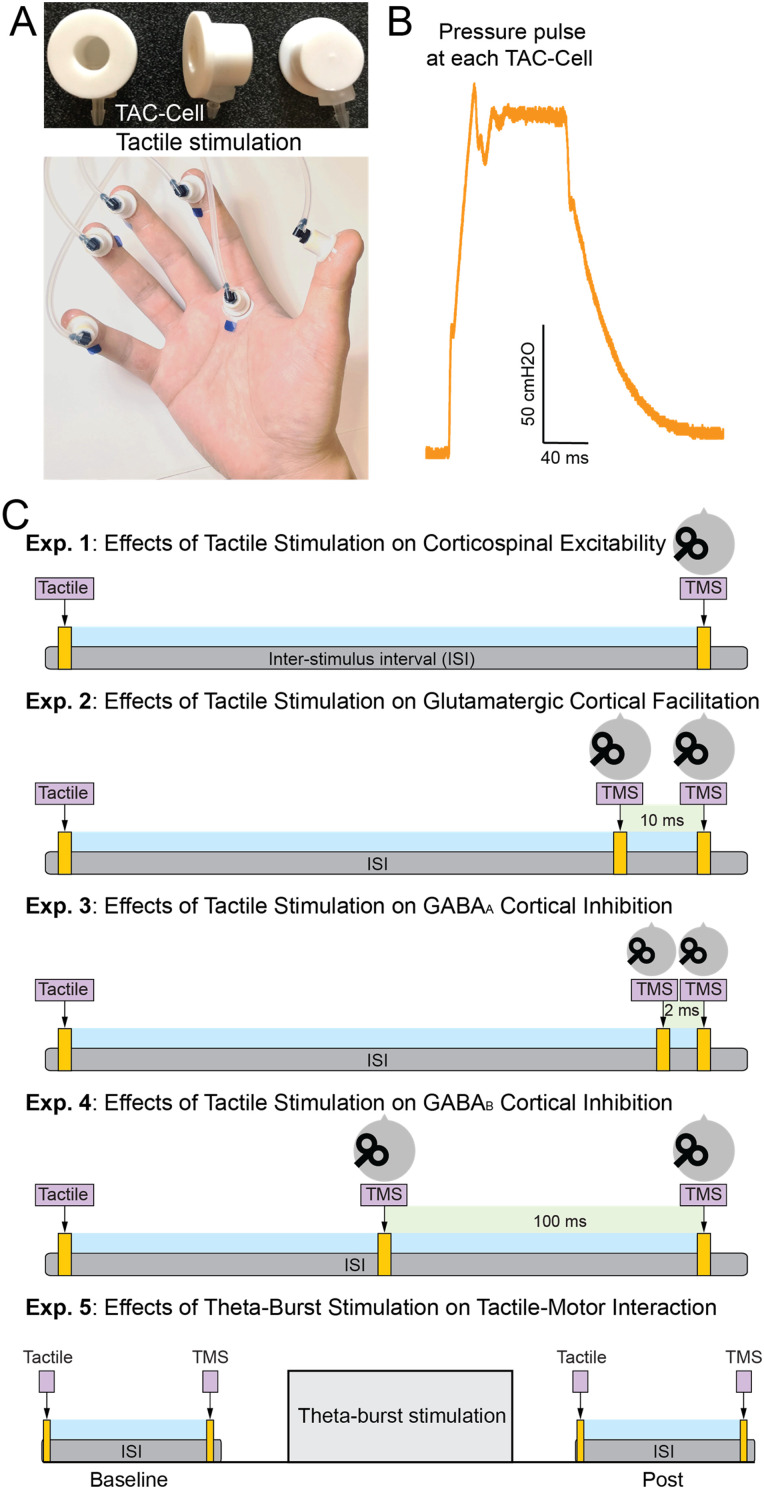
Experimental setup and protocols. ***A***, Tactile stimulation apparatus: display of air-filled plastic capsules used during the experiment (top panel) and demonstration of the tactile stimulation induced by air pressure (bottom panel). ***B***, The air pressure curve was applied to the hand's glabrous skin. ***C***, Tactile stimulation and TMS protocols across experiments: Exp. 1, tactile stimuli were applied to various parts of the hand, including the index fingertip, the base of the index finger, the ring fingertip, all five fingertips, and conditions without any stimulation. This was followed by TMS targeting the representation of the FDI muscle in the primary motor cortex (M1) of the opposite side. Exp. 2: the conditioning TMS was delivered 10 ms prior to the test TMS. The tactile stimuli were introduced 200 ms before the paired-TMS ICF condition. In Exp. 3: the conditioning TMS was applied 2 ms before the test TMS. Tactile stimulation was delivered 200 ms prior to the paired-TMS SICI condition. Exp. 4: the conditioning TMS was applied 100 ms before the test TMS. Tactile stimulation was delivered 200 ms prior to the paired-TMS LICI condition. Exp. 5: either cTBS or sham cTBS was administered to the primary somatosensory cortex (S1). The alterations in MEPs due to tactile stimulation were recorded both prior to and following the intervention.

We utilized a novel multichannel pneumotactile stimulation device (Epic Medical Concepts & Innovations) for this study. Pneumotactile stimulation elicits responses in somatosensory nerve fibers in a manner that resembles natural touch more closely (Hluštík et al., 2001). Tactile stimulation selectively activates rapidly adapting cutaneous mechanoreceptors without causing discomfort (Johansson and Vallbo, 1979). Conversely, electrical stimulation activates a broad spectrum of fibers with varied conduction velocities, affecting both the deeper and superficial receptors (Forss et al., 1994). When stimulating a mixed peripheral nerve electrically, it triggers both proprioceptive (mainly from muscle and joint receptors) and cutaneous (primarily from skin receptors) feedback to the CNS (Burke et al., 1988). Additionally, electrical stimulation can bypass certain skin receptors, especially those responsible for pressure transduction, and might be perceived as discomforting by participants (Rossini et al., 1996). To administer tactile stimulation, we affixed an array of miniature air-filled plastic capsules, termed TAC-Cells (6 mm in diameter), onto the glabrous skin of the right hand (Fig. 1*A*), using adhesive tape collars fortified with tincture of benzoin. Fabricated from acetal thermoplastic homopolymer, these TAC-Cells utilize tiny volumes of compressed air to rapidly deform the skin. Brief biphasic pressure pulses, lasting 80 ms with a force of 140 cmH_2_O, were applied to the hand's glabrous skin (Fig. 1*B*). The pneumotactile stimulation device provided a sharp rise time, generating highly synchronized tactile inputs, while ensuring no proprioceptive interference from any stimulus-induced finger joint movement. Participants characterized this sensation as “taps” or “raindrops” felt on their hands and reported no discomfort during the procedure.

### Transcranial magnetic stimulation

Magnetic stimulation of the brain was administered using a DuoMAG MP-Dual TMS system (Brainbox). The TMS coil was held tangentially on the scalp, angled 45° from the midline, with its handle pointing laterally and posteriorly [posterior-anterior (PA)–induced current in the brain]. This coil orientation, inducing a PA current flow, is recognized for effectively trans-synaptically activating M1 (Werhahn et al., 1994; Kaneko et al., 1996). We determined the optimal position by evoking the largest motor-evoked potential (MEP) in the FDI muscle (hotspot) with the minimum stimulus intensity (Rothwell et al., 1999). This hotspot was marked using a frameless neuronavigation system (Rogue Research) to maintain consistent coil placement throughout the study. The resting motor threshold (RMT) was determined as the minimum stimulus intensity that could generate an MEP exceeding 50 μV in peak-to-peak amplitude above the background EMG activity in at least 5 out of 10 consecutive trials (Rothwell et al., 1999).

### Study design

Participants were required to complete five main experiments across three separate visits (Fig. 1*C*). Specifically, during the first visit, Experiment 1 was conducted to examine the effects of tactile stimuli from single or multiple fingers on corticospinal excitability of the FDI muscle in a resting state. Following an interval of at least 1 d, Experiments 2–4 were conducted to examine the effects of tactile stimulation on ICF, SICI, and LICI. Note that only the tactile stimulation of the five fingertips was incorporated in Experiments 2–4. With another gap of at least 1 d, Experiment 5 was conducted to probe into the effects of S1 neuromodulation on tactile–motor interactions. Besides these five main experimental tasks, two control experiments related to Experiments 2–4 were carried out on a separate day.

### Experiment 1

While comfortably seated in a custom chair with both arms flexed at the elbow by 90°, participants were exposed to five potential stimulation types: index fingertip, base of the index finger, ring fingertip, all five fingertips, and no stimulation. The order in which these tactile stimuli were delivered was randomized, and participants were not informed beforehand about the specific type of stimulation they would encounter next. The impact of tactile stimulation on corticospinal excitability of the FDI muscle was assessed by applying tactile stimulation to the fingers, followed by TMS over the contralateral FDI representation in M1. The tactile stimulation was delivered at an interstimulus interval (ISI) of 200 before the TMS. To compute the changes in corticospinal excitability induced by each tactile stimulus (referred to as afferent inhibition), we compared the amplitude of the conditioned MEP (in the presence of tactile stimulation) with the amplitude of the test MEP (without tactile stimulation) using the equation: [(conditioned MEP × 100) / (test MEP)]. For each type of tactile stimulation, 15 test MEPs and 15 conditioned MEPs were recorded and analyzed. In a control experiment, we assessed the effect of tactile stimulation on the little finger and its subsequent effect on the corticospinal excitability of the FDI muscle.

### Experiment 2

We employed a paired-pulse TMS paradigm in conjunction with tactile stimulation to investigate the influence of tactile stimulation on ICF. ICF is linked to glutamate-mediated excitatory functions within M1 (Ziemann et al., 1998; Schwenkreis et al., 1999). During the assessment of ICF without tactile stimulation, TMS was applied to administer a conditioning stimulus (CS) at an intensity level of 80% of the RMT. The intensity for the test stimulus (TS) was established at a higher level, specifically 120% of the RMT. The CS preceded the TS by 10 ms. ICF was calculated by expressing the size of the conditioned MEP as a percentage of the size of the test MEP [(conditioned MEP × 100) / (test MEP)]. Fifteen test MEPs and 15 conditioned MEPs were tested. During the ICF assessment with tactile stimulation, the tactile stimulus was administered 200 ms prior to the TS in the ICF condition to investigate the potential modulatory impact of tactile stimulation on ICF. Recognizing that tactile stimulation might influence the MEP amplitude, a control experiment was tested by adjusting the MEP size to match MEP amplitudes in the nontactile ICF assessment.

### Experiment 3

SICI was used to make inferences about GABA_A_ receptor activity within M1 (Kujirai et al., 1993; Ziemann et al., 1996). The ISI for SICI was established at 2 ms. The CS intensity was configured at 70% of the RMT, while the TS intensity was established at 120% of the RMT. SICI was calculated by expressing the size of the conditioned MEP as a percentage of the size of the test MEP [(conditioned MEP × 100) / (test MEP)]. In the SICI assessment incorporating tactile stimulation, the tactile stimulus was applied 200 ms before the TS in the SICI condition to explore the potential modulatory effects of tactile input on SICI.

### Experiment 4

We employed the LICI protocol to examine GABA_B_ receptor-mediated activity within M1 (Valls-Solé et al., 1992; Wassermann et al., 1996). The intensity for the CS was configured at 120% of the RMT, and similarly, the TS was also set to an intensity level of 120% of the RMT. An ISI of 100 ms was selected for LICI measurements. LICI was calculated by expressing the size of the conditioned MEP as a percentage of the size of the test MEP [(conditioned MEP × 100) / (test MEP)]. To investigate the possible impact of tactile stimulation on LICI, we administered tactile input 200 ms prior to the TS in the LICI condition. Given the potential of tactile stimulation to influence the MEP amplitude, a control experiment was conducted in which the test MEP size was adjusted to match MEP amplitudes in the nontactile LICI assessment.

### Experiment 5

TBS can induce LTD- and LTP-like plasticity in the cortex (Huang et al., 2005). Extensive evidence indicates that cTBS predominantly decreases cortical excitability (Huang et al., 2005; Suppa et al., 2016; Rounis and Huang, 2020). Here, we administered cTBS to the S1 using a 70 mm figure-of-eight coil paired with a Magstim Rapid2 system (Magstim). The stimulation site for S1 was determined by measuring 2 cm posterior to the M1 hotspot using the neuronavigation system. Prior research has employed this approach to identify the effector homologous location in S1 that corresponds to M1 (Ishikawa et al., 2007; Conte et al., 2012; Tsang et al., 2014). Stimulation at S1 using this approach has been shown to modify the somatosensory-evoked potentials (SEPs; Wolters et al., 2005; Ishikawa et al., 2007). We delivered cTBS at an intensity of 70% RMT. The protocol consisted of 600 pulses at 30 Hz, separating bursts by a 167 ms interval (Gentner et al., 2008; Goldsworthy et al., 2014; Tsang et al., 2014; Mirdamadi and Block, 2021). A sham cTBS protocol was also administered over S1, with the coil oriented sideways on the scalp. Utilizing the afferent inhibition measurement, we examined the effects of S1 neuromodulation on tactile–motor interactions. Measurements were recorded at two separate intervals: 5 min before cTBS (establishing the baseline) and 5 min after cTBS.

### Data analysis

The Shapiro–Wilk's test was utilized to determine the normal distribution, while Levene's test of equality and Mauchly's test of sphericity assessed the homogeneity of variances. In instances where the normal distribution was not assumed, the data underwent a log transformation. If sphericity could not be assumed, the analysis relied on the Greenhouse–Geisser correction. In Experiment 1, involving 20 participants (*n* = 20), we utilized repeated-measures ANOVAs to assess the effects of different types of stimulation (the index fingertip, base of the index finger, ring fingertip, all five fingertips, and no stimulation) on corticospinal excitability and afferent inhibition. For detailed pairwise comparisons, we carried out a Bonferroni-corrected post hoc analysis. For Experiments 2–4, which involved 16 participants each, paired tests were used to determine the influence of tactile stimulation (with and without stimulation) on ICF, SICI, and LICI metrics. For Experiment 5 (*n* = 15), which included 15 participants, a repeated-measures ANOVA was conducted to determine the effects of the cTBS protocol (cTBS and sham cTBS) and the timeframe (baseline and post-cTBS) on afferent inhibition. Significance was set at *p* < 0.05.

## Results

### Specificity of single and multiple finger stimulation in modulating corticospinal excitability of the hand muscle

**Figure 2.**
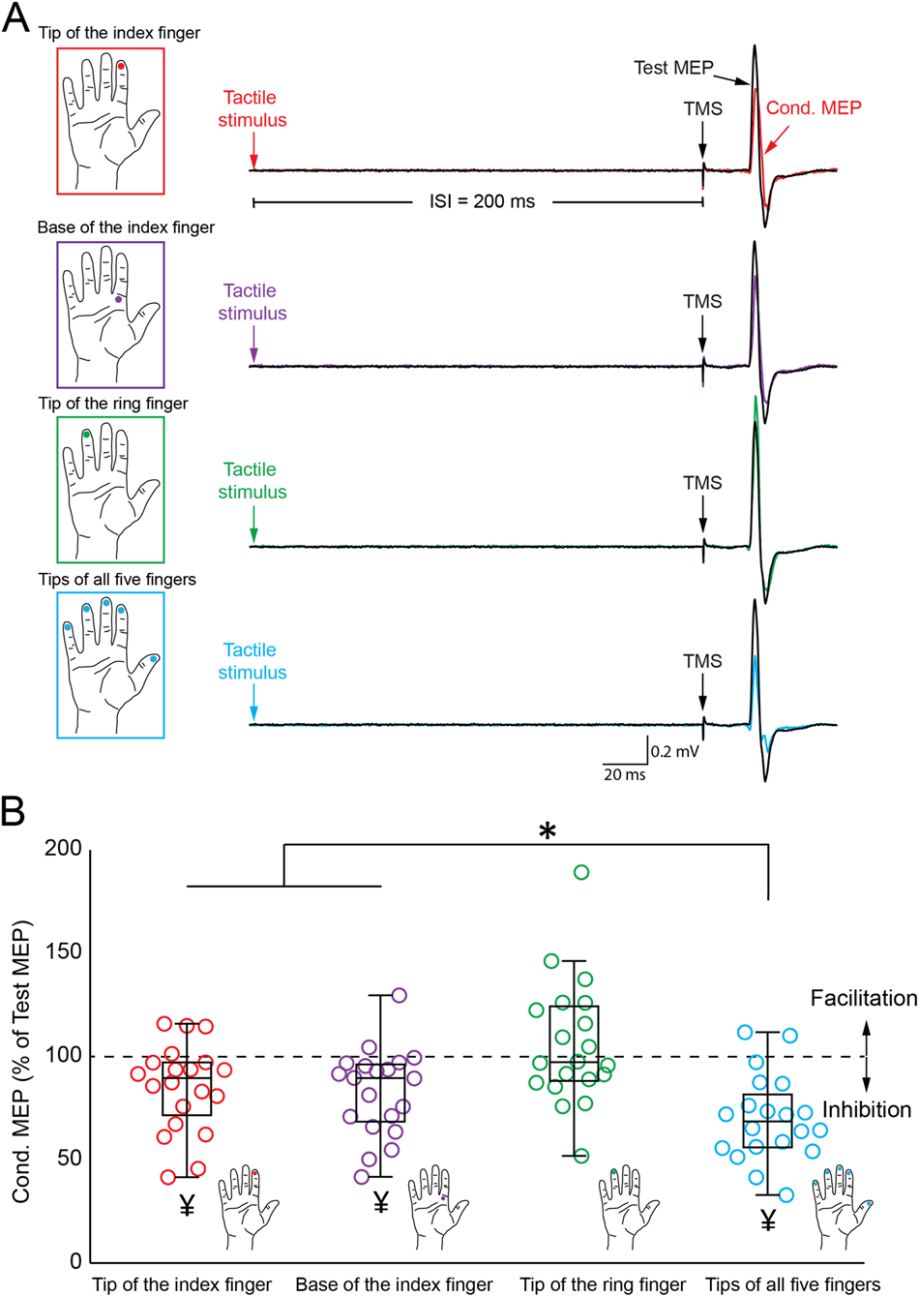
TMS-induced MEPs across all experiments. ***A***, The tactile stimulation sites for each experiment are depicted on the left panels, with corresponding representative MEP waveforms shown on the right. Black represents MEPs triggered by a single TMS pulse; colors represent MEPs induced by combined TMS and tactile stimuli. ***B***, The intensity of MEPs for four different stimulation sites (index fingertip, base of the index finger, ring fingertip, and all five fingertips) is illustrated using a box and whisker plot. MEP intensities from combined TMS and tactile stimulation are normalized to the single-pulse MEPs; values >100% denote a facilitation effect, while values <100% signify inhibition. The boxplot displays several elements: the bottom whisker denotes the minimum value, the first quartile marks the point below which 25% of the data falls, the median divides the dataset in half, the third quartile indicates the level below which 75% of the data lies, and the top whisker represents the maximum value. For added detail, individual data points are superimposed onto the boxplot. *p < 0.05 for comparisons between the cond. MEPs. ^¥^*p* < 0.05 for comparisons between the cond. MEPs and test MEP.

Tactile stimulation was administered via a pneumatic stimulator that exerted air pressure to indent the glabrous skin. The stimulation sites included the index fingertip, base of the index finger, ring fingertip, all five fingertips, or no stimulation was given. Figure 2*A* illustrates the examples of test and conditioned MEPs recorded from the FDI muscle of a representative subject. Note that the conditioned MEPs were suppressed compared with the test MEPs when tactile stimulation was applied to the index fingertip (indicated in red), the base of the index finger (in purple), or all five fingertips (in blue). Stimulation of a single finger, whether the index fingertip or base, resulted in less suppression of conditioned MEPs than when all five fingers were stimulated. Despite the higher innervation density of the fingertip compared with the finger's base (Corniani and Saal, 2020), both stimulations showed comparable conditioned MEP suppression. On the other hand, heterotopic (ring finger) tactile stimulation (in green) did not lead to any suppression of the conditioned MEPs when compared with the test MEP.

Repeated-measures ANOVA revealed a significant effect of tactile stimulation (*F*_(4,76)_ = 12.014; *p* < 0.001) on corticospinal excitability, as evidenced by the amplitude changes in the MEPs. In the post hoc analysis, the α level was adjusted to 0.0125 in accordance with the Bonferroni’s correction. This adjustment involved dividing the α value of 0.05 by four to account for the four distinct pairwise comparisons in our study: index fingertip stimulation versus no stimulation, base of the index finger stimulation versus no stimulation, ring fingertip stimulation versus no stimulation, and stimulation of all five fingertips versus no stimulation. The results indicated that the amplitudes of MEPs were reduced in conditions involving stimulation over the index fingertip (MEP = 0.82 ± 0.38 mV; *p* = 0.008), the base of the index finger (MEP = 0.81 ± 0.44 mV; *p* = 0.001), and all five fingertips (MEP = 0.67 ± 0.32 mV; *p* < 0.001), compared with the MEP amplitudes in the condition where no tactile stimulation was applied (MEP = 0.97 ± 0.38 mV). The amplitude of MEPs showed no significant difference between the condition involving ring finger stimulation and the condition where tactile stimulation was absent (*p* = 0.9). Repeated-measures ANOVA revealed a significant effect of different stimulation types (*F*_(3,57)_ = 13.246; *p* < 0.001) on afferent inhibition, calculated as [(conditioned MEP × 100) / (test MEP)]. For the subsequent post hoc analysis, the α level was set at 0.0167, following the Bonferroni’s correction for the three pairwise comparisons. This adjustment was made by dividing the alpha value of 0.05 by three, accommodating the three distinct pairwise comparisons which involved assessing afferent inhibition with stimulation of the index fingertip, the base of the index finger, and all five fingers. Stimulation of all five fingertips led to more marked afferent inhibition (70.2 ± 20.7%) compared with either the index fingertip (85.2 ± 21.1%; *p* = 0.005) or the base of the index finger (82.8 ± 21.0%; *p* = 0.003). However, no significant difference in afferent inhibition was found between stimulation of the index fingertip and its base (*p* = 0.6). Afferent inhibition was not observed with stimulation of the ring fingertip (105.9 ± 30.0%). Similarly, in the control experiment, no afferent inhibition occurred when stimulating the tip of the little finger (106.7 ± 14.5%; *p* = 0.3).

### Finger stimulation increases ICF

**Figure 3.**
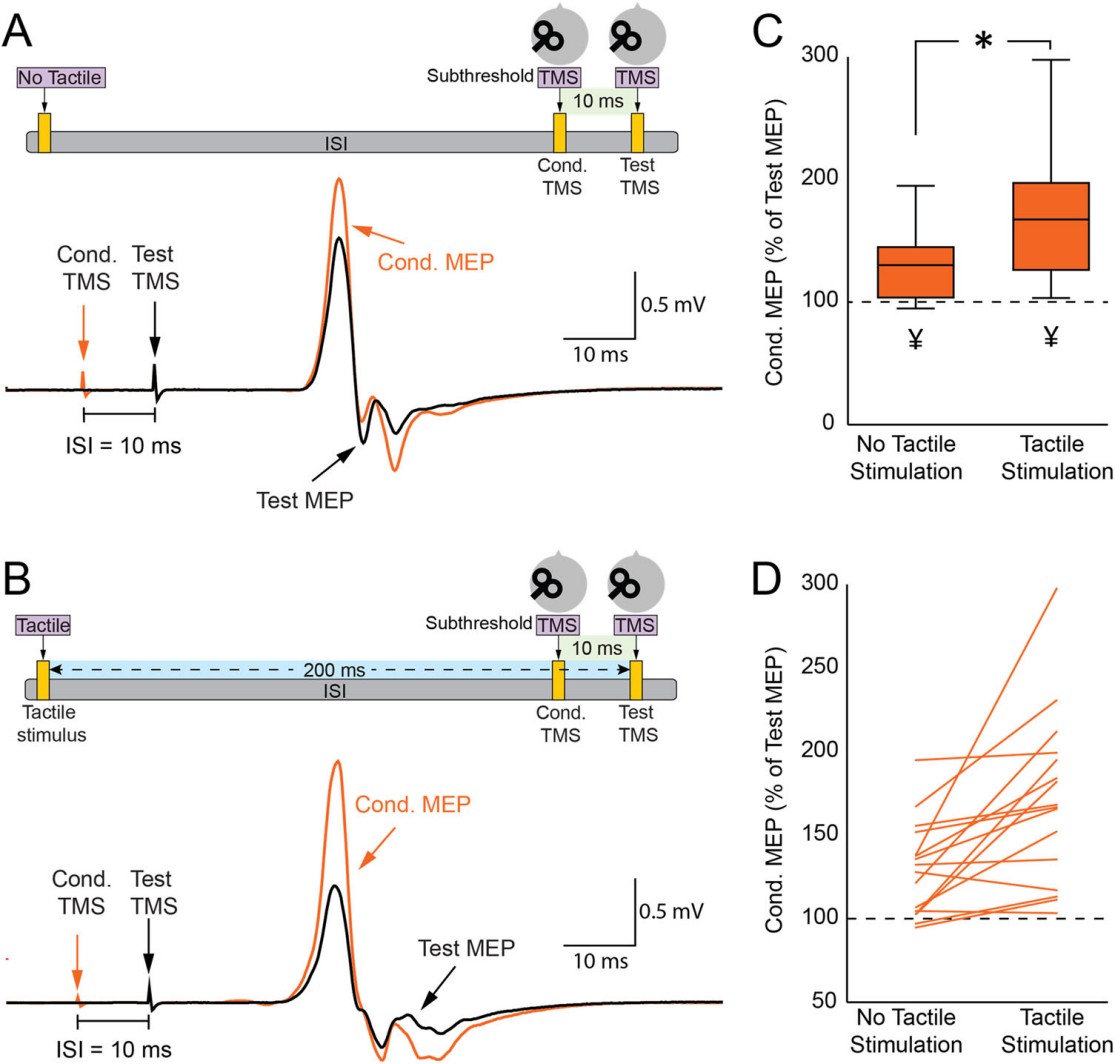
Tactile stimulation influences ICF. ***A***, Top, ICF setup: the conditioning TMS preceded the test TMS by 10 ms. Bottom, MEPs elicited by TMS with (red) and without (black) the conditioning TMS. ***B***, Top, ICF setup including tactile stimulation. Bottom, MEP waveforms from paired (red) and single (black) TMS stimuli when tactile stimulation was given 200 ms before the TS in the ICF condition. ***C***, Box and whisker plot displaying ICF with and without tactile stimulation. ***D***, Individual changes in ICF across both stimulation conditions. *p < 0.05, comparing ICF with and without tactile stimulation; ^¥^*p* < 0.05, comparing test MEP with conditioned MEP.

Figure 3*A* displays the test MEP (in black) and the conditioned MEP (in orange) recorded from the FDI muscle during the ICF assessment using the paired-pulse TMS paradigm. Note that the conditioned MEP was facilitated compared with the test MEP when a subthreshold conditioned TMS was delivered to M1 10 ms prior to the test TMS over M1. The paired *t* test showed an elevation in the amplitudes of conditioned MEPs (2.19 ± 0.76 mV; *p* < 0.001) in comparison with the amplitudes of the test MEPs (1.75 ± 0.68 mV), thereby confirming the presence of ICF. Meanwhile, Figure 3*B* shows the test MEP (in black) and the conditioned MEP (in orange) during the integration of tactile stimulation into the ICF assessment. The conditioned MEP exhibits enhanced facilitation compared with the test MEP when tactile stimulation is administered 200 ms prior to the paired-TMS ICF protocol. The results from the paired *t* test indicated that ICF with tactile stimulation (170.9 ± 51.0%) was significantly greater than ICF without tactile stimulation (129.4 ± 28.2%; *p* = 0.002; Fig. 3*C*), implying that tactile stimulation notably augmented ICF. As further evidence, the majority of participants (14 out of 16) exhibited increased ICF when tactile stimulation was introduced (Fig. 3*D*). Based on the results from Experiment 1, which showed that tactile stimulation reduced the MEP amplitude, we performed a control experiment. In this experiment, the MEP size in the ICF with tactile stimulation was adjusted to match the MEP amplitudes observed in the ICF assessment without tactile stimulation. Despite this adjustment, our results continued to demonstrate that ICF with tactile stimulation was significantly larger than ICF without tactile stimulation (*p* = 0.03).

### Finger stimulation does not affect SICI

**Figure 4.**
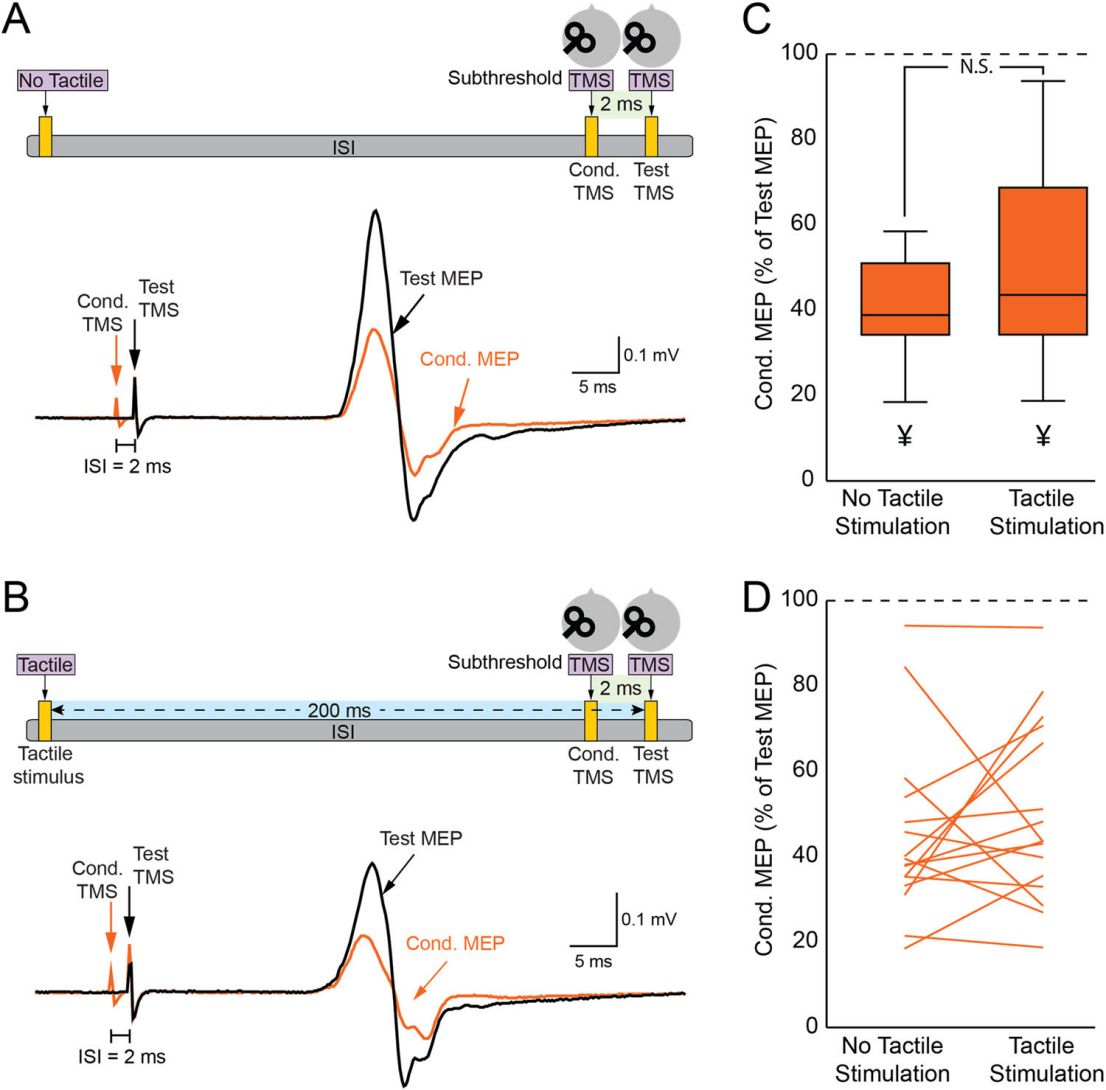
Tactile stimulation does not influence SICI. ***A***, SICI setup with depicted MEPs: tactile stimulation was not applied, and the ISI between the CS and TS was 2 ms. MEPs from the paired-pulse TMS are less intense than those from single-pulse TMS. ***B***, SICI setup when tactile stimulation was involved, delivered 200 ms before the TS in the SICI condition. The bottom panel showcases representative MEP waveforms. ***C***, Box and whisker plot comparing SICI in the presence and absence of tactile stimulation. ***D***, Individual variations in SICI for each participant across both conditions. ^¥^*p* < 0.05, comparing test MEP with conditioned MEP.

Figure 4*A* depicts the test MEP (in black) and conditioned MEP (in orange) recorded from the FDI muscle during the SICI assessment. When a subthreshold conditioned TMS was administered to M1 2 ms before the test TMS over M1, the conditioned MEP exhibited a notable inhibition in comparison with the test MEP. The paired *t* test revealed a reduction in the amplitudes of conditioned MEPs (0.73 ± 0.37 mV; *p* < 0.001) in comparison with the amplitudes of the test MEPs (1.67 ± 0.61 mV), thereby confirming the occurrence of SICI. Figure 4*B* presents the test MEP (in black) and the conditioned MEP (in orange) when tactile stimulation was incorporated into the SICI assessment. Note that the extent of inhibition observed during SICI with tactile stimulation was comparable with SICI without tactile stimulation. Further analysis using a paired *t* test showed that the extent of SICI with tactile stimulation (49.7 ± 21.1%) was not significantly different from SICI without tactile stimulation (44.7 ± 20.3%; *p* = 0.4; Fig. 4*C,D*), suggesting that tactile stimulation did not modulate SICI.

### Finger stimulation decreases LICI

**Figure 5.**
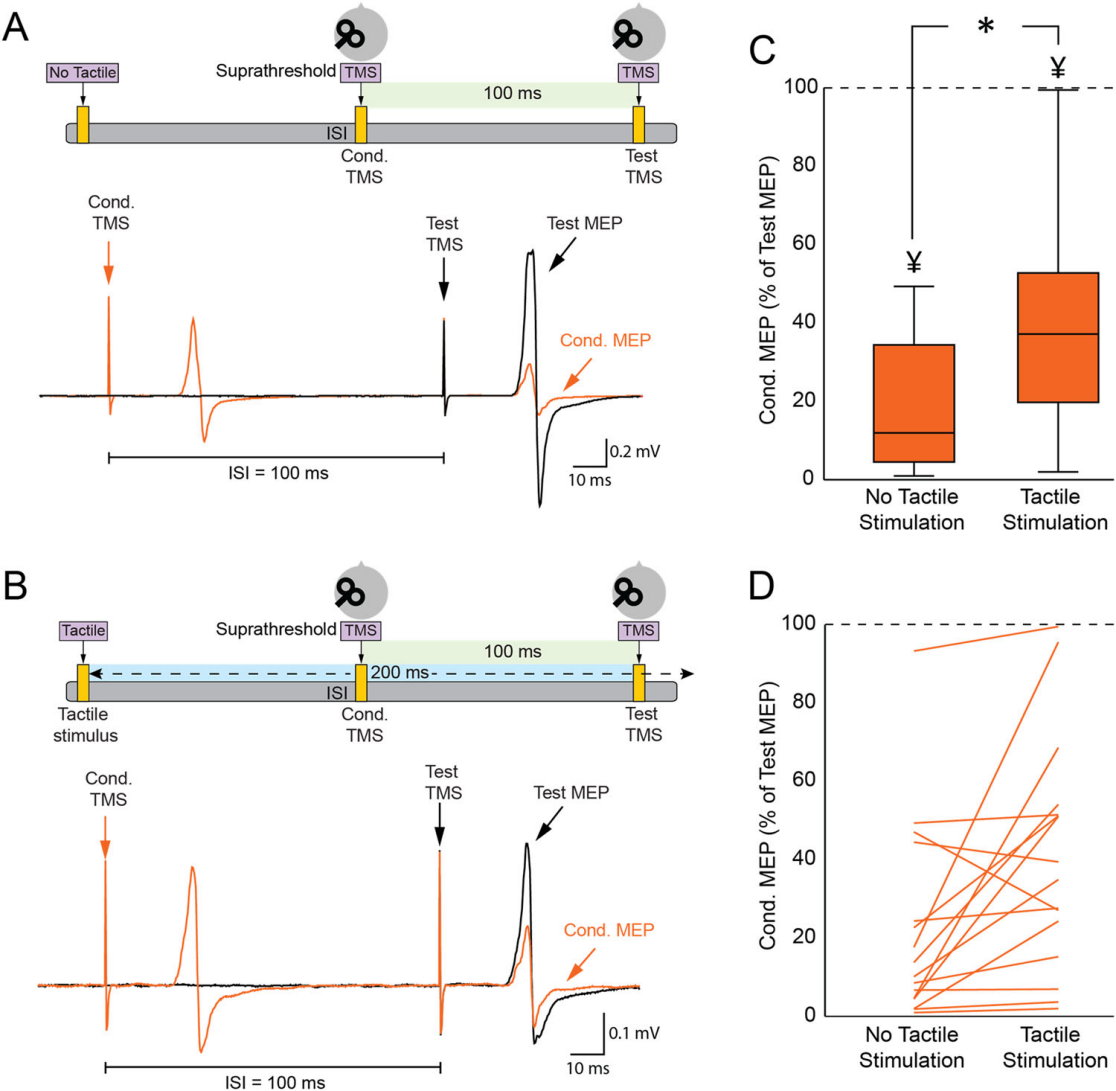
Tactile stimulation influences LICI. ***A***, LICI setup with illustrated MEPs: the ISI between the CS and TS was set at 100 ms. Given the CS intensity exceeded the RMT, an FDI MEP followed the CS. The MEP amplitude from the paired-pulse TMS was reduced compared with the single-pulse TMS. ***B***, LICI setup when tactile stimulation was introduced, delivered 200 ms prior to the TS in the LICI condition. The bottom panel displays representative MEP waveforms. ***C***, Box and whisker plot contrasting LICI in conditions with and without tactile stimulation. ***D***, Individual LICI variations for each participant in both tactile and nontactile conditions. *p < 0.05, comparing LICI in the presence and absence of tactile stimulation; ^¥^*p* < 0.05, comparing test MEP with conditioned MEP.

Figure 5*A* shows the test MEP (in black) and the conditioned MEP (in orange) during the LICI assessment utilizing the paired-pulse TMS paradigm. The conditioned MEP was suppressed in comparison with the test MEP when a suprathreshold conditioned TMS was administered to M1 100 ms before the test TMS over M1. The paired *t* test indicated a decrease in the amplitudes of conditioned MEPs (0.44 ± 0.75 mV; *p* < 0.001) compared with the amplitudes of the test MEPs (1.74 ± 0.65 mV), thus demonstrating LICI's presence. Figure 5*B* illustrates the test MEP (in black) and conditioned MEPs (in orange) when tactile stimulation was integrated into the LICI assessment. When tactile stimulation preceded the paired-TMS LICI protocol by 200 ms, the conditioned MEP exhibited reduced inhibition compared with the test MEP. The paired *t* test results revealed that LICI under tactile stimulation (40.8 ± 29.5%) was significantly reduced compared with LICI in the absence of tactile stimulation (22.0 ± 25.1%; *p* = 0.01; Fig. 5*C*), suggesting that tactile stimulation significantly diminished LICI. Highlighting this, a major proportion of the participants (14 out of 16) presented with a decrease in LICI upon the addition of tactile stimulation (Fig. 5*D*). Given Experiment 1's results, in which tactile stimulation reduced the MEP amplitude, a control experiment was conducted. In this, the MEP size during tactile LICI was adjusted to match the MEP amplitudes from the nontactile LICI assessment. Our results showed that LICI with tactile stimulation was significantly decreased compared with nontactile LICI (*p* = 0.02).

### S1 neuromodulation affect tactile–motor interactions

**Figure 6.**
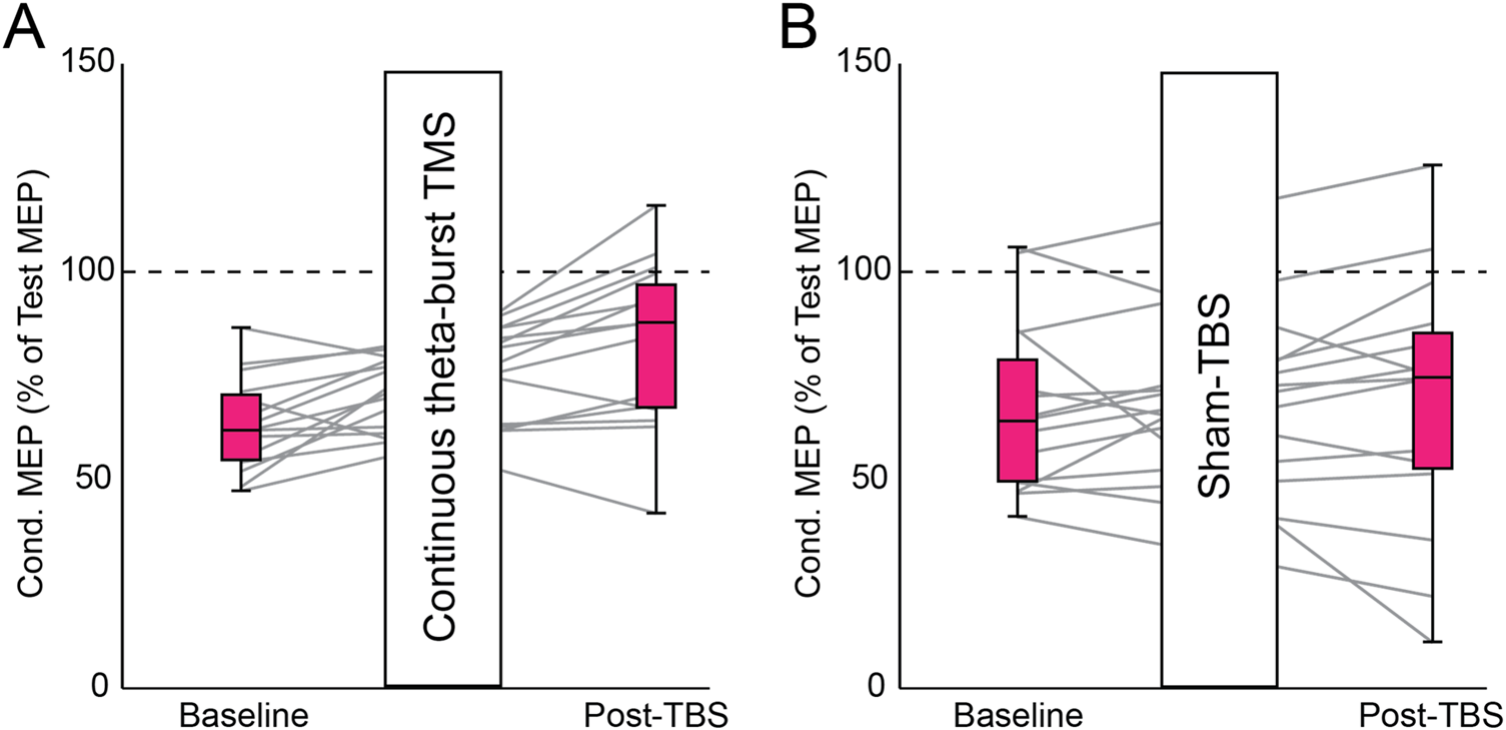
cTBS versus sham cTBS: the magnitudes of test MEPs (following tactile stimulation) were normalized to the conditioned MEPs without tactile stimulation. The box and whisker plot displays the median, quartiles, and extreme values, with the gray lines representing individual participant data. Panels ***A*** and ***B*** show results from cTBS and sham cTBS, respectively.

Figure 6, *A* and *B*, presents the group data delineating the temporal progression of alterations in afferent inhibition during cTBS and sham procedures. Observably, post-cTBS over S1, there was a noticeable reduction in afferent inhibition relative to the baseline. Conversely, following sham stimulation, the level of afferent inhibition remained similar before and after the procedure. To ascertain if cTBS over S1 influenced M1 excitability, we evaluated the MEP amplitude without tactile stimulation both pre- and post-cTBS. Our analysis revealed no significant difference in MEP amplitude before cTBS over S1 (0.95 ± 0.31 mV) compared with that after cTBS (1.00 ± 0.36 mV; *p* = 0.6). The repeated-measures ANOVA revealed significant effects of the timeframe (*F*_(1,28)_ = 4.541; *p* = 0.04) on afferent inhibition. Subsequent post hoc analyses revealed that post-cTBS, afferent inhibition significantly reduced to 82.9 ± 19.8% from the baseline of 63.5 ± 11.3% (*p* = 0.01). After the sham stimulation, the level of afferent inhibition poststimulation (68.9 ± 30.9%) was consistent with the baseline (67.1 ± 20.5%; *p* = 0.8).

## Discussion

In this study, we systemically examined the contributions of tactile inputs from the glabrous skin of the hand to corticospinal excitability of the hand muscles and the activities within M1's intracortical circuits. Our findings demonstrate that tactile sensations from the hand's glabrous skin shape corticospinal excitability of the hand muscles, contingent on the spatial characteristics of tactile inputs. When tactile stimulation was administered to a finger homotopic to the target muscle, regardless of the specific location of stimulation (fingertip or base), there was a decrease in corticospinal excitability. In contrast, tactile stimuli to a finger distant from the target muscle did not elicit the same response. Notably, simultaneous stimulation of all fingers yielded a more significant reduction in corticospinal excitability of the muscle than individual finger stimulation. Additionally, tactile inputs considerably augmented ICF and reduced LICI without affecting SICI. We also found that downregulating S1 excitability via cTBS affected tactile–motor interactions. We suggest that the spatial specificity of tactile inputs in shaping corticospinal excitability might facilitate the execution of skilled hand movements. Furthermore, we propose that tactile inputs distinctly modulate the excitatory and inhibitory circuits within M1, laying the foundation for tactile–motor interaction, with S1 playing a crucial role in this interaction.

One of the standout features of our experimental methodology involved the use of a multichannel pneumatic stimulator. This device was designed to activate the LTMRs by delivering controlled volumes of air-driven mechanical force. Touch sensation from the glabrous skin of the hand is derived from mechanosensory structures that convert mechanical pressures exerted on the skin into electric impulses, which is subsequently transmitted to the CNS via the axons of LTMRs (Handler and Ginty, 2021). Contrary to the electrical finger stimulation employed in prior research, our tactile stimulation approach elicits responses in somatosensory nerve fibers that more closely mimic the sensation of actual touch (Hluštík et al., 2001). Notably, in our investigation, homotopic (index finger) tactile stimulation—and not heterotopic (ring finger) tactile stimulation—triggered afferent inhibition in the FDI muscle. This aligns with prior research indicating that the magnitude of afferent inhibition induced by electrical finger stimulation is contingent upon the spatial correlation between the stimulated finger and the hand muscle targeted by TMS (Tamburin et al., 2001; Di Lazzaro et al., 2005; Dubbioso et al., 2017). Given that sensory input often arises from movement, it is likely that homotopic afferent inhibition is influenced by our hand usage. Research indicates that the ring finger frequently comoves with other fingers to facilitate grasping (Ingram et al., 2008), with its enslaved force exceeding that of the index finger (Yu et al., 2010). Conversely, the index finger typically exhibits a greater degree of independent movement (Ingram et al., 2008). As highlighted by Ejaz et al. (2015), the way we utilize our hands dictates the organization of finger-specific activity patterns within the sensorimotor cortex. To explore this further, we executed a control experiment assessing whether heterotopic (little finger) tactile stimulation diminished corticospinal excitability of the FDI muscle, given that the little finger typically operates more independently and its enslaved force is less than that of the ring finger. Our results showed that tactile stimulation of the little finger did not induce afferent inhibition, indicating that the manner of hand usage might not be a factor in our findings. Moreover, our findings highlighted that the simultaneous stimulation of all five fingers led to a more pronounced reduction in corticospinal excitability of the FDI muscle compared with individual finger stimulation. This observation aligns with the findings from human imaging studies on multifinger stimulation (Arbuckle et al., 2022) and animal research concerning multiwhisker stimulation (Laboy-Juárez et al., 2019). These studies suggest that somatosensory inputs from multiple fingers or whiskers result in a unique neural representation. Notably, Arbuckle et al. (2022) revealed that stimulating multiple fingers results in increased interactions within S1 and M1, involving inputs from fingers that are both homotopic and heterotopic. Echoing these findings, we posit that when tactile inputs from multiple fingers are present, the corticospinal excitability of hand muscles is modulated by inputs from all these fingers.

The human fingertip is densely packed with tactile afferent fibers, reaching up to 141 units/cm^2^, in contrast to the reduced density of 25 units/cm^2^ found at the finger's base (Corniani and Saal, 2020). The higher innervation density at the fingertip leads to enhanced tactile sensitivity compared with the finger's base (Johansson and Vallbo, 1983). Intriguingly, despite this marked difference in innervation density between the index fingertip and its base, our research reveals that tactile sensations from the index fingertip reduce corticospinal excitability of the FDI muscle to a similar extent as stimulation at the base of the index finger. There are two plausible explanations for this observation. Firstly, the pathway of somatosensory information flows is VPL → S1 → M1 (Sherman, 2016; Jabaudon, 2017; Jung et al., 2021), indicating that VPL and S1 process and possibly filter out irrelevant tactile inputs before reaching M1 (Lei and Perez, 2017; Lei et al., 2018). Thus, it might not be the quantity but rather the quality of tactile information that influences the corticospinal excitability of the muscle. Secondly, previous studies have indicated no significant relationship between a region's tactile innervation density and its cortical representation size (Corniani and Saal, 2020). This implies that the impact of tactile inputs on corticospinal excitability is perhaps more associated with the somatotopic arrangement of the anatomical links between S1 and M1, rather than the density of tactile innervation.

Within M1, complex interactions occur between various intracortical facilitatory and inhibitory circuits. Evidence indicates that the effect of somatosensory inputs on corticospinal excitability is mediated through interactions with intracortical excitatory and inhibitory interneurons within M1, which is essential for regulating corticospinal excitability (Sailer et al., 2002; Udupa et al., 2014; Cash et al., 2015). In our Experiments 2–4, we explored the impact of tactile inputs from the hand's glabrous skin on distinct excitatory and inhibitory mechanisms in M1: glutamate-mediated ICF, GABA_A_-mediated SICI, and GABA_B_-mediated LICI. Our results indicated that tactile inputs notably augmented ICF and diminished LICI. However, SICI remained unaffected by these tactile inputs. We speculate that excitatory tactile inputs project on the excitatory interneurons associated with ICF within M1, amplifying ICF (Cash et al., 2015). Simultaneously, these inputs appear to suppress the inhibitory GABA_B_ activities responsible for LICI. Our findings align with previous studies, in which ICF was significantly increased (Cash et al., 2015) and LICI was decreased (Sailer et al., 2002; Udupa et al., 2014) in the presence of afferent inhibition. It is noteworthy that tactile inputs did not affect SICI in our study, which aligns with the findings of Sailer et al. (2002). However, this stands in contrast to the results presented by Udupa et al. (2014). A key factor that might explain this difference is the latency between tactile stimulation and SICI. In our study, as well as in the study by Sailer et al. (2002), tactile stimuli were administered 200 ms prior to SICI. Given an ISI of 200 ms, afferent inhibition likely has a cortical origin, as spinal cord excitability remains unaffected (Chen et al., 1999; Classen et al., 2000). Conversely, Udupa et al. (2014) employed afferent inhibition latencies of 23 or 25 ms, suggesting a potential spinal origin for this inhibition (Caccia et al., 1973; Tarkka and Larsen, 1986; Uncini et al., 1991). Moreover, the modulation of muscle activity by cutaneous reflexes (Nakajima et al., 2006; Zehr et al., 2014), elicited through direct stimulation of the skin, further supports the notion of a spinal contribution to the modulation of corticospinal excitability. These findings underscore the complexity of the neural mechanisms involved and highlight the interplay between cortical and spinal pathways in shaping motor responses.

Tactile–motor interaction is believed to be mediated through S1, as evidenced by research revealing that S1 neuromodulation can alter corticospinal excitability (Jacobs et al., 2014). The essential role of S1 in processing tactile information is well established (Romo et al., 2012), and its capability to modulate M1 excitability in response to somatosensory inputs is mediated through a cortical pathway from SI to M1 (Classen et al., 2000). Our previous studies found a concurrent increase in MEPs within the ipsilateral M1 and a decrease in the SEP within ipsilateral S1 during motor activities (Lei and Perez, 2017), underscoring the important interplay between M1 and S1. Furthermore, research has indicated that hand areas of M1 identified via electrical brain stimulation align with areas exhibiting pronounced N20 SEP peaks (Fukuda et al., 2008), implying shared neural circuits between M1 and S1. In Experiment 5, we examined the influence of S1 on tactile–motor interactions by employing cTBS to suppress S1 neural activity. After applying cTBS to S1, we found a significant decrease in afferent inhibition compared with the baseline. We propose that cTBS over SI diminishes the responsiveness of SI to tactile inputs. A decrease in tactile inputs to S1 would reduce its modulation of M1, leading to a drop in afferent inhibition. Our results align with previous studies that demonstrated cTBS over S1 led to a decrease in high-frequency oscillations within the SI (Ishikawa et al., 2007), impaired tactile acuity (Rai et al., 2012), and diminished short-latency afferent inhibition (Tsang et al., 2014). Thus, we suggest that S1 plays an important role in integrating tactile signals from the hand's glabrous skin into corticospinal excitability.

In conclusion, our study highlights the significant influence of tactile feedback from the hand's glabrous skin in modulating corticospinal excitability of hand muscles. Our findings reveal that tactile stimuli substantially alter the excitatory and inhibitory pathways within M1, indicating a cortical origin for the modulation of corticospinal excitability. The potential contribution of subcortical regions to this modulation is an area that remains to be explored and will be the focus of future investigations. Our research suggests that the primary somatosensory cortex (S1) is integral in the convergence and processing of tactile and motor signals.

## Data Availability

The data that support the findings of this study are available from the corresponding author upon reasonable request.

## Synthesis

Reviewing Editor: David Franklin, Technische Universitat Munchen Decisions are customarily a result of the Reviewing Editor and the peer reviewers coming together and discussing their recommendations until a consensus is reached. When revisions are invited, a fact-based synthesis statement explaining their decision and outlining what is needed to prepare a revision will be listed below. The following reviewer(s) agreed to reveal their identity: Keiichi Kitajo. Note: If this manuscript was transferred from JNeurosci and a decision was made to accept the manuscript without peer review, a brief statement to this effect will instead be what is listed below.

Your manuscript has now been reviewed by two experts; one of whom reviewed your manuscript previously. We all think that the manuscript highlights important findings regarding the mapping of tactile stimuli onto motor representations, extending previous observations. However, we all believe that the current version of the manuscript requires additional analysis, and additional description of the current analysis to fully support the findings. I encourage you to revise the manuscript according to the comments from the two reviewers.

Reviewer 1:

The authors have carefully addressed my comments and clarified several of my previous concerns. Overall, this revised version of the manuscript provides clearer information and useful insight that may advance our knowledge of how tactile stimuli guide motor behaviour.

There remain a few points that the authors may still address to further improve their manuscript:

1) In the discussion, the sentence "We propose that the neural representation of tactile inputs from multiple fingers reflects the interactions of individual fingers, which might explain the reduced corticospinal excitability induced by five-finger stimulation compared to single-finger stimulation" (429-432) seems to outreach the scope of the study and not fully supported by the data. Indeed, the study points at the mapping of tactile inputs onto the corticospinal system, rather than (more generally) at their neural representation, that can be quite different across different parts of the sensorimotor cortex, as shown in Arbuckle et al. 2022 (cited in the manuscript). The authors may consider rephrasing this passage and/or discussing better this issue (e.g., they may refer more narrowly to the motor representation of tactile stimuli).

2) It is true that the pinkie is usually controlled in a more independent fashion compared to the ring finger, as shown in the literature cited in the manuscript (e.g., Yu et al., 2009, Ingram et al., 2008). However, both these fingers have a similar functional relationship with the index finger, especially compared to the thumb (see Ejaz et al., 2015). In this regard, while the new control experiment with the pinkie helps clarifying how tactile stimuli are mapped onto M1, it hardly supports the conclusion that "the manner of hand usage might not be a factor in our findings". For example, it could be that stimulation delivered to the thumb influences corticospinal excitability measured from the FDI, owing to the frequent co-usage of the thumb and the index finger (e.g., pinching movements).

3) I am now more convinced that cTBS over S1 did not affect M1 excitability. In the new version of the manuscript, MEPs amplitude pre and post cTBS are reported in their absolute value in mV. One additional control that could be easily performed is to normalize MEPs amplitude in each participant to 0 mean, 1 SD (i.e., z-score). This should eliminate inter-individual variability (which is usually quite large in MEPs) and highlight any pre/post difference.

4) In several points throughout the manuscript the authors refer to the influence of tactile stimuli on the corticospinal output. I assume here TMS was delivered at rest, although this does not seem to be explicitly specified in the methods - authors should add this important methodological information in the manuscript. If this is the case, then in my view this terminology is inappropriate. When TMS pulses are delivered at rest, the corticospinal output (in this case, to the FDI) remains necessarily the same across different conditions. What TMS measures is rather the excitability of the corticospinal projections directed towards the FDI. In fact, corticospinal output (or activity) and excitability are two quite different physiological phenomena that can be modulated independently (see Matthews, 1994, The effect of firing on the excitability of a model motoneurone and its implications for cortical stimulation, J Physiol), as in the case of the current data where a significant modulation of excitability occurs while the corticospinal output remains null.

5) Minor observation: at line 386, the index finger is defined "adjacent" to the FDI muscle. Perhaps it may be better to use here the same terminology employed throughout the manuscript (homo/heterotopic)

Reviewer 2:

The authors explored the modulation of motor cortical inhibition and facilitation by tactile stimulation on the glabrous skin of the fingers. They utilized an advanced air-puff paradigm combined with intracortical facilitation (ICF), long-interval cortical inhibition (LICI), short-interval cortical inhibition (SICI), and continuous theta-burst stimulation (cTBS) techniques. While the study's design appears robust, there are significant concerns regarding the statistical results. The lack of detailed statistical analyses and results makes it difficult to support the study's conclusions convincingly.

On page 14

>Note that the conditioned MEPs were suppressed compared to the test MEPs when >tactile stimulation was applied to the index fingertip (indicated in red), the base of

> the index finger (in purple), or all five fingertips (in blue).

Regarding Experiment 1, it's unclear if the authors performed any statistical analyses pertinent to this particular aspect. How did the authors compare the test-only MEPs and conditioned MEPs? While Figure 2 includes p-values for comparisons between finger conditions, there is a noticeable absence of detailed information regarding the differences between test-only and conditioned MEPs. I would appreciate if the authors could provide comprehensive results from the ANOVA and post-hoc tests to clarify this matter

Given the presence of four finger conditions, it is imperative to address the issue of multiple comparisons to ascertain if air-puff conditioned MEPs are significantly lower than test MEPs. I suggest the authors consider applying a correction for multiple comparisons. Furthermore, the methodology for post-hoc comparisons between conditioned MEPs across different finger conditions, particularly in the context of multiple comparison correction, is not clearly detailed. I would appreciate the inclusion of specific information regarding the post-hoc analyses in the method section.

On page 15, regarding ICF

>Note that the conditioned MEP was facilitated compared to the test MEP when a >subthreshold conditioned TMS was delivered to M1 10 ms prior to the test TMS over >M1, indicating the presence of ICF.

The presentation of statistical results related to the differences between conditioned and non-conditioned motor evoked potentials (MEPs) in the ICF paradigm is notably absent. Demonstrating these statistical differences is crucial to validate the efficacy of the ICF paradigm. Additionally, I request detailed statistical analyses for the other paradigms, including SICI and LICI. The report lacks comprehensive details on the outcomes of these paradigms, underscoring the need for a thorough presentation of the statistical analyses.

The explanation of the boxplots in the study requires further clarification. The authors should explicitly define what each component represents, such as the mean, median, 75th percentile, maximum, and minimum values, to enhance clarity. Additionally, including individual data points overlaid on the boxplots would provide more informative and detailed insights.

On page 10

> When introducing tactile stimulation to the ICF assessment, tactile stimulation was

> applied 200 ms before the paired-TMS ICF condition, thereby exploring the potential >modulatory effects of tactile stimulation.

The description of the timing for tactile stimulation is unclear - it's not specified whether it was administered 200ms before the conditioning stimulus (CS) or the test stimulus (TS). This ambiguity also extends to the descriptions for ICF, LICI, and other similar paradigms. I recommend that these sections and figure captions be revised for clearer delineation of the timing of tactile stimulation in relation to both the CS and TS.

On page 12 ,

>The stimulation site for S1 was determined by measuring 2 cm

>posterior to the M1 hotspot using the neuronavigation system.

If an MRI-based neuronavigation system was not utilized in the study, the term 'neuronavigation' should be omitted. Simply positioning the coil 2cm posterior to the M1 hotspot does not constitute the use of a neuronavigation system.
